# Environmental & load data: 1:15 Scale tidal turbine subject to a variety of regular wave conditions

**DOI:** 10.1016/j.dib.2019.103732

**Published:** 2019-03-07

**Authors:** S. Draycott, G.S. Payne, J. Steynor, A. Nambiar, B. Sellar, T. Davey, D.R. Noble, V. Venugopal

**Affiliations:** a School of Engineering, Institute for Energy Systems, The University of Edinburgh, Edinburgh, EH9 3DW, UK; b Naval Architecture, Ocean and Marine Engineering, University of Strathclyde, Glasgow, G4 0LZ, UK

## Abstract

Experimental data was obtained in order to investigate the effect of waves on the loads and performance of tidal turbines. An instrumented 1:15 scale tidal turbine was installed in the FloWave Ocean Energy Research Facility, and a wide range of regular wave conditions were generated; systematically varying both wave frequency and height. Waves were generated both following and opposing a fixed mean current velocity of 0.81 m/s. Data are made available of the measured turbine loads and environmental conditions obtained for five repeats of 24 wave conditions via https://doi.org/10.7488/ds/2472. A description of the data collection process, data processing, file structure and naming conventions are provided in this article. The analysis and presentation of the described dataset can be found in Ref. [1].

**Table:** Specifications table

Subject area	*Engineering*
More specific subject area	*Tidal stream turbines*
Type of data	*Tables, Text files (.txt)*
How data was acquired	***Environmental:****surface elevations measured using a resistance wave gauge, and current velocities (U, V, W) using a Nortek Vectrino Profiler.* ***Loads:****AMTI OR6-7 load-cell mounted on the test tank floor used to measure 6DOF loads on the whole turbine and structure. Turbine-installed-instrumentation (rotor thrust, torque and position) acquired using bespoke transducers and an encoder.*
Data format	*Raw text files (unfiltered, no QC conduced)*
Experimental factors	*All instrumentation calibrated prior to testing, with the wave gauge calibrated daily. Each morning zero load measurements are taken, and removed, for all instruments prior to testing.*
Experimental features	*A 1:15 scale tidal turbine is subject to combined wave-current conditions. These conditions are generated in a circular combined wave & current test tank: The FloWave Ocean Energy Research Facility (*www.flowave.eng.ed.ac.uk*). The measured conditions, and corresponding loads measured on the turbine are provided.*
Data source location	*Edinburgh, UK (Latitude: 55.922048 Longitude: -3.178620)*
Data accessibility	*The data is available at*https://doi.org/10.7488/ds/2472
Related research article	*S. Draycott, G. Payne, J. Steynor, A. Nambiar, B. Sellar, and V. Venugopal, “An experimental investigation into non-linear wave loading on horizontal axis tidal turbines,” J. Fluids Struct., vol. 84, pp. 199–217, 2019.*https://doi.org/10.1016/j.jfluidstructs.2018.11.004

**Table:** 

**Value of the data** • This large open-source dataset will facilitate increased understanding into the nature and extent of wave-induced loads on tidal stream turbines: informing design and control requirements. • These data can be used to validate numerical models such as those based on Blade Element Momentum Theory (BEMT) codes or Computational Fluid Dynamics (CFD). • This extensive benchmark dataset can also be used for comparison to other tidal stream turbines in similar conditions, or to the same turbine design in different conditions. This can facilitate increased understanding of inter-turbine and inter-facility differences.

## 1 Data

The data described in this article was obtained from experimental tank testing of a 1:15 scale tidal stream turbine subject to a range of regular wave conditions in the presence of a fixed mean current velocity. Waves are generated both following and opposing the current direction, and wave frequencies and amplitudes are systematically varied. Turbine load data, along with corresponding surface elevations and velocity measurements are provided. Files are available for download at https://doi.org/10.7488/ds/2472 and consist of text files for five repeats of 24 wave conditions. Three files are provided per test, partitioned by the data-type (and sample frequency): surface elevation (128 Hz), fluid velocity (100 Hz) & turbine loads (256 Hz). The file-naming convention is detailed in Section 2.5, referring to Table 1, Table 2, Table 3, Table 4, Table 5.

**Table 1:** Wave height file identifiers.

Wave height ID	H1	H2	H3	H4	H5	H6	H7	H8
Relative wave height	0.25	0.5	1	1.5	1.75	2	2.25	4

**Table 2:** Wave frequency file identifiers.

Wave frequency ID	F1	F2	F3	F4	F5	F6
Wave frequency [Hz]	0.308	0.348	0.4	0.444	0.5	0.545

**Table 3:** Wave direction file identifiers.

Wave Direction ID	O	F
Wave direction	opposing	following

**Table 4:** Data type file identifiers.

DAQ ID	WG	ADV	LOADS
Channels	wave gauge	*U,V, W*	*T, Q,* θ*, Fx, Fy, Fz, Mx, My, Mz*

**Table 5:** Repeat number file identifiers.

Repeat ID	R1	R2	R3	R4	R5
Repeat number	1	2	3	4	5

## 2 Experimental design, materials and methods

### 2.1 The turbine and set-up

The Tidal Stream Turbine (TST) model is a three-bladed horizontal axis machine with a diameter of 1.2 m and is nominally a 1:15 scale representation of a generic horizontal-axis tidal turbine. The design of the turbine is covered in detail in Ref. [2], with CAD drawings and baseline turbine data available for download at https://doi.org/10.7488/ds/1707 [3]. It should be noted these CAD files correspond to an earlier version of the turbine with a 76mm shorter nacelle beyond the tower. On-board sensors measure thrust, *T,* torque, *Q*, and angular position, θ.

In addition to sensors integrated into the model tidal turbine, additional instruments were installed throughout the FloWave Ocean Energy Research Facility (see e.g. Refs. [4], [5], [6], for recent work assessing combined wave-current conditions). The location and types of instruments are detailed in Draycott et al. (2019) [1] (table 1). Environmental conditions are measured using a resistance-type wave gauge to measure water surface elevation, *ƞ,* and an Acoustic Doppler Velocimeter (ADV) to measure current velocity in the *x, y* and *z* directions (*U, V, W*). Additional load measurements are obtained using a bottom-mounted six-axes (6-DOF) load cell: measuring forces, *F*, and moments, *M*, on the entire TST structure (blades + TST body + tower). The reader is referred to Ref. [1] (Fig. 4) for a diagrammatic representation of the test layout.

### 2.2 Test conditions

For all tests the mean current velocity (with no turbine present) is kept at 0.81 m/s. Regular waves are generated both following and opposing the mean current direction; varying both wave frequency and height. Six wave frequencies are generated for following waves (waves propagating in the same direction as the mean current) and three for opposing waves, as described in Ref. [1] (tables 3 and 4). For every frequency-direction combination initially wave heights of 0.1 m are targeted using an iterative correction procedure. Once correct within an acceptable tolerance, additional wave amplitudes are obtained using a linear gain applied to these corrected wave amplitudes for two of the wave frequencies (0.308 Hz and 0.4 Hz). These gain values were chosen to assess the effect of wave amplitude on the turbine loads with consideration of the limits of the wave paddles and load sensors.

### 2.3 Data collection

#### 2.3.1 Instrument operation and calibration

The various instruments used in this test programme were operated and calibrated as follows. The AMTI 6DOF loadcell uses a “smart amplifier” with an internally stored calibration matrix (factory supplied). Six analogue outputs ( ±10V) are then supplied to the data acquisition system (DAQ) with preset linear calibration functions. Similarly, the ADV has an internal stored factory calibration. The model of ADV used in these tests does not incorporate analogue outputs and the data is logged on a PC using proprietary software. The wave gauges were calibrated daily using a minimum of three measurement points. Wave gauges are logged on the main tank control PC and are synchronised by an internal trigger to the wave maker operation. An external trigger (5V pulse) is provided to provide synchonisation to the DAQ and ADV system.

The combined torque and thrust sensor internal to the turbine is operated using its factory supplied calibration. The root bending moment sensors are calibrated using a variable lever arm with multiple weights up to a maximum of approximately 100Nm.

The calibration functions are applied internally within the DAQ with calibrated and raw values logged. The digital inputs from the motor encoder are processed using counters on a Field Programmable Gate Array (FPGA) within the DAQ to give position and rotational velocity (RPM) outputs.

#### 2.3.2 DAQ architecture

**Fig. 1:**
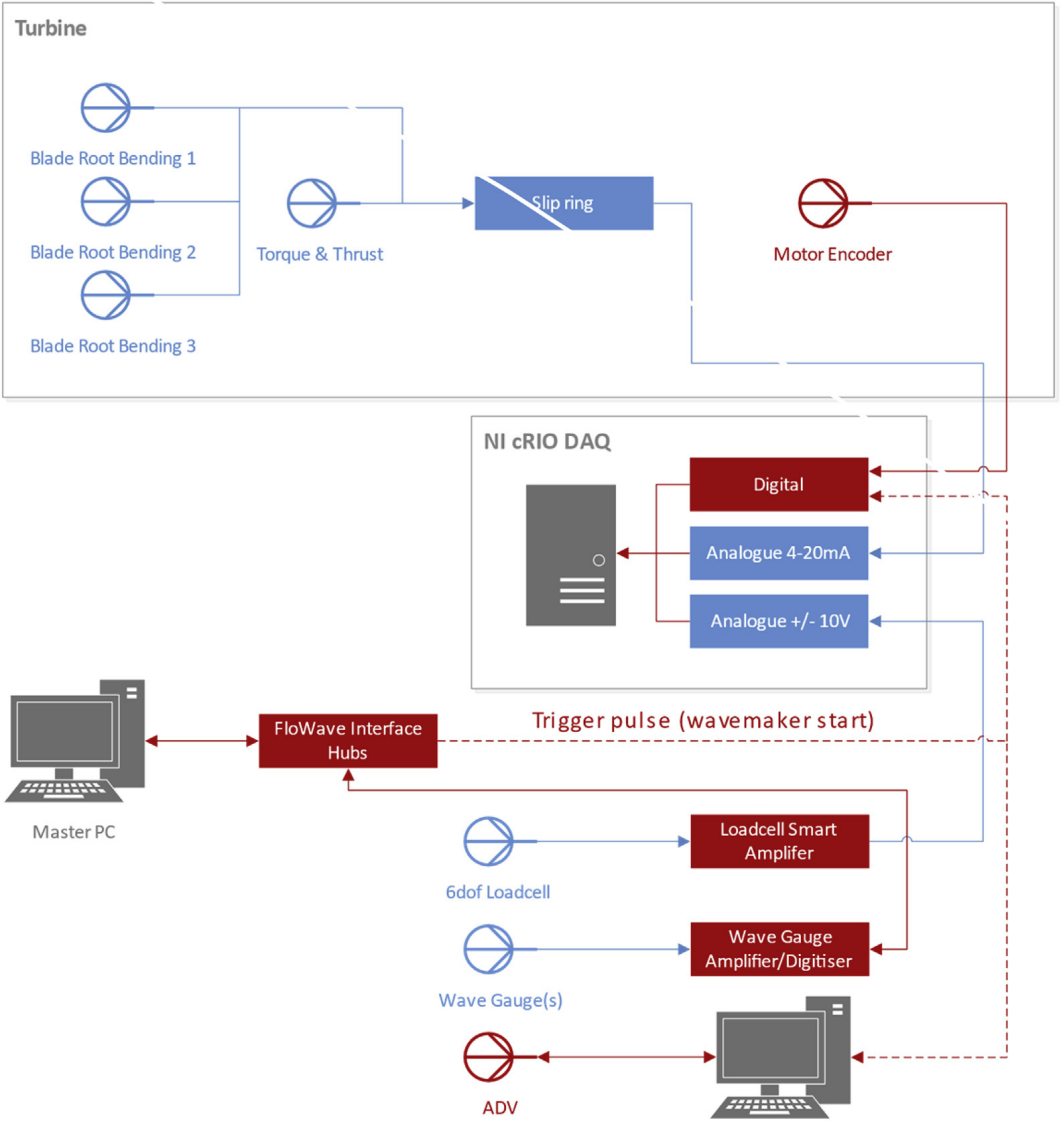
Data acquisition architecture.

The primary DAQ used within this experimental programme was a National Instruments cRIO-9082 modular system. All signals from the turbine itself and the 6DOF loadcell are processed and logged on this system. The signals from the wave gauges and ADV are logged on separate proprietary software and are synchonised to the start of wavemaker operation using a common trigger signal. The architecture and general arrangement of the acquisition system is illustrated in Fig. 1.

### 2.4 Data processing

No filtering, smoothing or Quality Control (QC) has been applied to the data provided. The data has, however, been cropped to only include good quality data where the conditions are stable, and no wave reflections are present. The test segments provided align with the analysis presented in Ref. [1]. The start and end times were chosen predominantly using a visual assessment, with an upper time limit enforced based on the estimated group velocities; ensuring wave reflections could not have arrived at the model location. For a given frequency-direction combination (all amplitudes) the cropped test times are fixed.

### 2.5 File details and structure

As there are different sample frequencies for each of the instrument types (see Section 1) there are three files created for every test. All data is provided in SI units. The specifics of the test are given in each filename as follows:

[Wave height ID]_[Frequency ID]_[Wave Direction ID]_[DAQ ID]_[Repeat ID]

The identifiers are defined below:

As an example, for the opposing wave condition with a relative wave height of 1, and a frequency of 0.4 Hz. The name of the file containing the load data for the third repeat would be:H3_F3_O_Loads_R3.txt
